# Coenzyme Q10 in the eye isomerizes by sunlight irradiation

**DOI:** 10.1038/s41598-022-16343-8

**Published:** 2022-07-15

**Authors:** Md. Al Mamun, Md. Mahamodun Nabi, Tomohito Sato, Shuhei Aramaki, Yusuke Takanashi, Takumi Sakamoto, Kaito Hizume, Chikako Mori, Maiha Yasue, Masataka Ozaki, Ariful Islam, Tomoaki Kahyo, Makoto Horikawa, Yutaka Takahashi, Shigetoshi Okazaki, Kentaro Ohishi, Yu Nagashima, Keiji Seno, Yoshihiro Hotta, Mitsutoshi Setou

**Affiliations:** 1 Department of Cellular and Molecular Anatomy, Hamamatsu University School of Medicine, 1-20-1 Handayama, Higashi-ku, Hamamatsu, Shizuoka 431-3192 Japan; 2 Department of Radiation Oncology, Hamamatsu University School of Medicine, 1-20-1 Handayama, Higashi-ku, Hamamatsu, Shizuoka 431-3192 Japan; 3 First Department of Surgery, Hamamatsu University School of Medicine, 1-20-1 Handayama, Higashi-ku, Hamamatsu, Shizuoka 431-3192 Japan; 4 Department of Molecular Biotechnology, Graduate School of Advanced Science of Matter, Hiroshima University, Higashi-Hiroshima, Hiroshima 739-7763 Japan; 5 HAMAMATSU BioPhotonics Innovation Chair, Institute for Medical Photonics Research, Preeminent Medical Photonics Education and Research Center, Hamamatsu University School of Medicine, 1-20-1 Handayama, Higashi-ku, Hamamatsu, Shizuoka 431-3192 Japan; 6 Institute for Medical Photonics Research, Preeminent Medical Photonics Education and Research Center, Hamamatsu University School of Medicine, 1-20-1 Handayama, Higashi-ku, Hamamatsu, Shizuoka 431-3192 Japan; 7 Department of Biology, Hamamatsu University School of Medicine, 1-20-1 Handayama, Higashi-ku, Hamamatsu, Shizuoka 431-3192 Japan; 8 Department of Ophthalmology, Hamamatsu University School of Medicine, 1-20-1 Handayama, Higashi-ku, Hamamatsu, Shizuoka 431-3192 Japan; 9 International Mass Imaging Center, Hamamatsu University School of Medicine, 1-20-1 Handayama, Higashi-ku, Hamamatsu, Shizuoka 431-3192 Japan; 10 Department of Systems Molecular Anatomy, Institute for Medical Photonics Research, Preeminent Medical Photonics Education and Research Center, 1-20-1 Handayama, Higashi-ku, Hamamatsu, Shizuoka 431-3192 Japan

**Keywords:** Medical research, Lipids, Lipids, Eye diseases

## Abstract

Photoisomerization of lipids has been well studied. As for the eyes, photoisomerization from 11-*cis* isomer to all-*trans*-retinal is well-known as the first step of the visual transduction in the photoreceptors. In addition to that, there would be other ocular lipids that undergo photoisomerization, which may be involved in ocular health and function. To explore any photoisomerizable lipids in the eyes, the nonirradiated and sunlight-irradiated eyeball extracts were subjected to liquid chromatography-mass spectrometry analysis, followed by the identification of the decreased lipid species in the irradiated extracts. Surprisingly, more than nine hundred lipid species were decreased in the irradiated extracts. Three lipid species, coenzyme Q10 (CoQ10), triglyceride(58:4), and coenzyme Q9, were decreased both significantly (*p* < 0.05) and by more than two-fold, where CoQ10 showed the most significant decrease. Later, photoisomerization was identified as the prominent cause underlying the decrease of CoQ10. Interestingly, CoQ10 in the sunlight-irradiated fresh eyeballs was also isomerized. Both the visible light and ultraviolet radiation were capable of producing CoQ10 isomer, while the latter showed rapid action. This study is believed to enhance our understanding of the biochemistry and photodamage of the eye and can potentially contribute to the advancement of opto-lipidomics.

## Introduction

Photoisomerization is a photochemical process that produces isomer(s) of a molecule, often accompanied by a change in physical and chemical properties, upon light absorption^1,2^. In the medical field, the altered physical properties of photoisomers have been exploited to develop a therapeutic strategy. A prominent example is noninvasive phototherapy for the treatment of jaundice^3^. In this strategy, blue light illumination converts bilirubin to the photoisomeric form, changes its solubility and enhances its discharge from the body of the jaundice patient^4^. In recent years, photoswitchable molecules also have emerged as a photochemical tool to manipulate as well as control cell signaling optically^5,6^. In this way, technologies related to the photoisomerization of biomaterials can be applied in wide-ranging fields, from life science to medicine.

Among biomaterials, lipids are promising candidates that have the potential to undergo isomerization by environmental stimuli (such as sunlight exposure) and can exert altered biological functions. Lipids are hydrophobic or amphipathic biomolecules that show surprisingly higher diversity in their chemical structures. They are the major structural components of biological membranes, and the changes in their composition are involved in the pathogenesis of numerous diseases, including cancers^7^. An important aspect of lipid species is that they can exist in numerous structural and stereoisomers due to the presence of double bonds and various functional groups in their structure^8^. In the living organism, the biological function of a lipid molecule depends on its particular isomeric state. It is known that the *cis* geometry of the polyunsaturated fatty acid (PUFA)-containing lipids is essential for maintaining membrane homeostasis; hence, their specific geometry is strictly controlled by the enzymatic action^9^. Another example is conjugated linoleic acid, which has several isomer-specific biological effects, including anti-carcinogenic effects^10^. In addition, photoisomerization of lipids may lead to their dysregulation, and it is therefore important to deeply understand the biochemical characteristics of photoisomerized lipids. However, the production, distribution, and biochemistry of those isomeric lipids are largely unexplored.

One of the most well-studied photoisomerization reactions is the photoisomerization of 11-*cis* retinal in the photoreceptors of the eye. Such retinoids are classified into prenol lipids^11^ along with carotenoids and coenzyme Q10 (CoQ10). The eyes are the sensory organ that detects light and provides vision through a process known as the visual cycle^12^. The cycle primarily involves the *cis–trans* photoisomerization of the retinal chromophore, which is covalently bound to G-protein coupled receptors called opsins. In the presence of light, 11-*cis* retinal undergoes photoisomerization to all-*trans*-retinal that activates the opsin leading to a signal transduction cascade^12^.

In addition to the retinoids, other lipids are also known to play a crucial role in vision. It has been reported that the retina contains a highly rich amount of very long-chain polyunsaturated fatty acid (VLC-PUFA)-containing glycerophospholipids, which are synthesized by the enzyme elongation of very-long-chain fatty acid-4 (ELOVL4). Harkewicz et al. demonstrated that ELOVL4 expression is essential for VLC-PUFA synthesis and retinal functions^13^. Oxidation and the degradation of lipids in the eyes by light exposure are well-documented^14^. However, the isomerization of lipids in this organ due to irradiation has not been studied extensively. Here, we aimed to explore the photoisomerizable lipid species in the eyes that could help us understand the biochemistry and photodamage of this organ.

In the current study, we revealed that CoQ10 in the eyeball extracts and fresh eyeballs undergoes photoisomerization by sunlight irradiation. CoQ10 (also called ubiquinone-10) has a chain structure composed of ten isoprene units attached to a quinone head group. It is the most common form of the coenzyme family in humans and plays a crucial role in the electron-transport chain of mitochondria. It is an important lipid-soluble antioxidant that inhibits the oxidation of various biomolecules^15^ and plays a role in anti-inflammation^16^ and anti-apoptosis^17^. Supplementation of CoQ10 has been shown to have beneficial effects in numerous disorders, including Parkinson’s disease^18^, obesity^19^, and diabetes mellitus^20^. In this paper, we found the photoisomerization of CoQ10 under sunlight irradiation and therefore discussed the characteristics of the CoQ10 isomer.

## Results

### Exploration of potential photoisomerizable lipids in the eyeball

**Figure 1:**
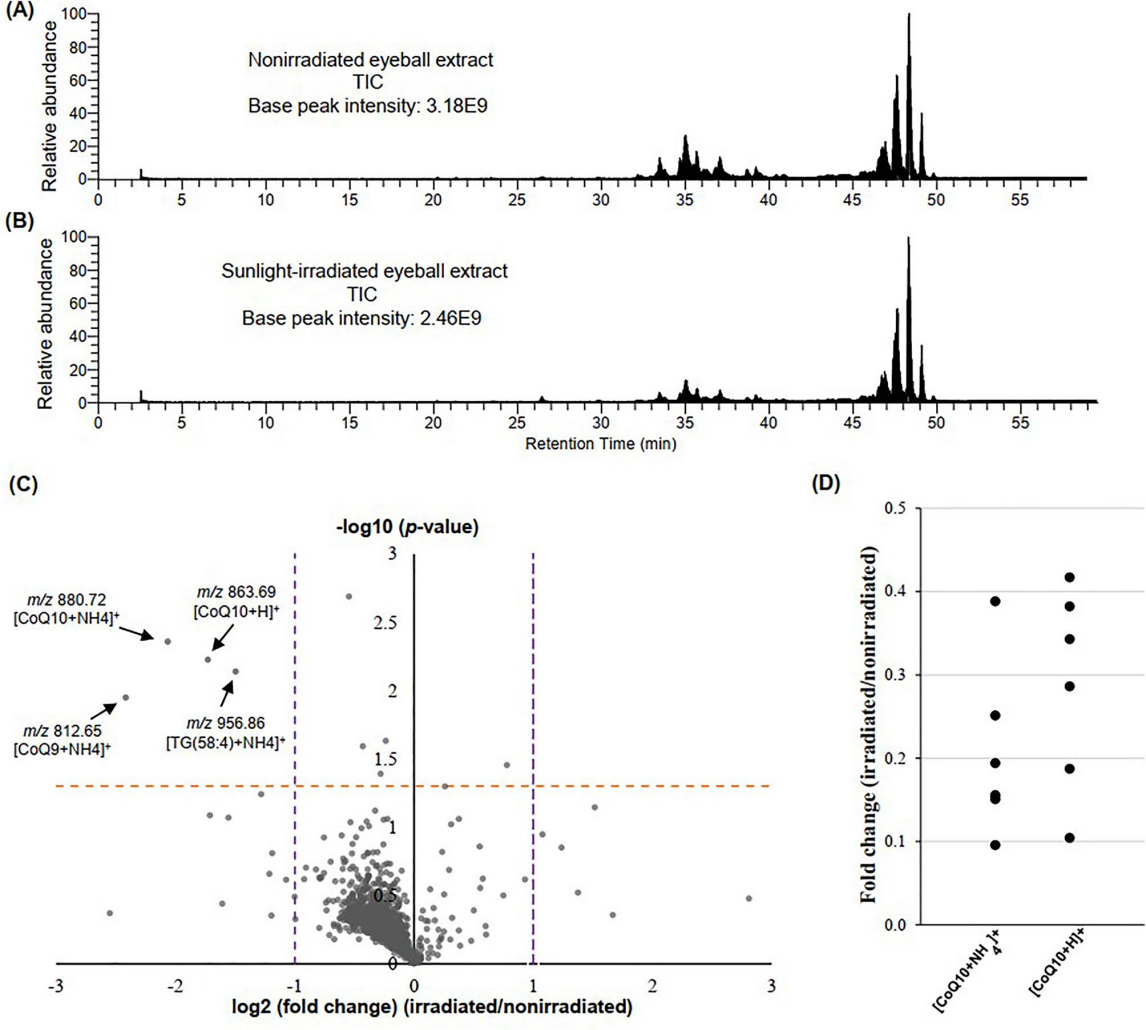
Exploration of potential isomerizable lipids in eyeballs upon sunlight irradiation. Total ion chromatogram (TIC) was obtained from the (**A**) nonirradiated and (**B**) irradiated eyeball extract (data of the first paired sample was shown here). Both positive and negative ion mode data were shown in the TIC. (**C**) Volcano plots of 1001 identified lipid species in the eyeball extracts. The dashed orange line show where *p* = 0.05. The dashed purple lines at the right and left show where fold change (irradiated/nonirradiated) = 2 and 0.5, respectively. The ions-of-interest that showed both large magnitude fold changes (x-axis) and high statistical significance (y-axis) were annotated. (**D**) Distributions of fold changes (irradiated/nonirradiated) of ions associated with CoQ10. A single dot in the dot plot indicates the fold change of one paired sample. The average retention time for [M + H]^+^ and [M + NH_4_]^+^ of CoQ10 shown here was 45.69 min and 45.70 min, respectively. *CoQ10* Coenzyme Q10, *CoQ9* Coenzyme Q9, *TG*(*58:4*) triglyceride(58:4).

We irradiated six paired eyeball lipid extracts in the sunlight for over a day in order to explore any photoisomerizable lipids in the eyeball (Fig. S1). Interestingly, the light irradiation decreased the darkness of the extract (Fig. S1). The total ion chromatogram (TIC) obtained from liquid chromatography-mass spectrometry (LC/MS) analysis showed a similar pattern in nonirradiated and irradiated extracts (Fig. 1A,B). To explore the potential photoisomerizable lipid molecules, we first identified the decreased molecular species by volcano plot analysis. Surprisingly, the volcano plot showed a decrease of more than nine hundred lipid-associated ions (including both positive and negative ions) in the sunlight-irradiated samples (Fig. 1C, Supplementary file 1). Of these, four positive ions at *m/z* 880.72, 863.69, 956.86, and 812.65 were decreased both significantly (*p* < 0.05) and by more than two-fold in irradiated samples compared to the nonirradiated ones (Fig. 1C). The first two ions (*m/z* 880.72 and 863.69) were tentatively assigned as [M + NH_4_]^+^ and [M + H]^+^ adduct of CoQ10 (oxidized), respectively (Fig. 1C, Table S1, Supplementary file 1). The other two ions (*m/z* 956.86 and 812.65) were associated with [M + NH_4_]^+^ adduct of triglyceride (58:4) and coenzyme Q9, respectively (Fig. 1C, Table S1, Supplementary file 1). In the current study, we further focused on CoQ10, which may have the strong potentiality of isomerization, as it showed the most significant decrease among those three lipids of interest. The dot plot showed that both ions (protonated and ammonium adduct) associated with CoQ10 decreased (ranging between 2 to 10-folds) in all six paired eyeball extracts upon sunlight irradiation (Fig. 1D). It is important to note that the reduced form of CoQ10 (CoQ10H2) was not detected in the LC/MS data of the eyeball extract sample.

### Assignment of CoQ10 in eyeball extract

**Figure 2:**
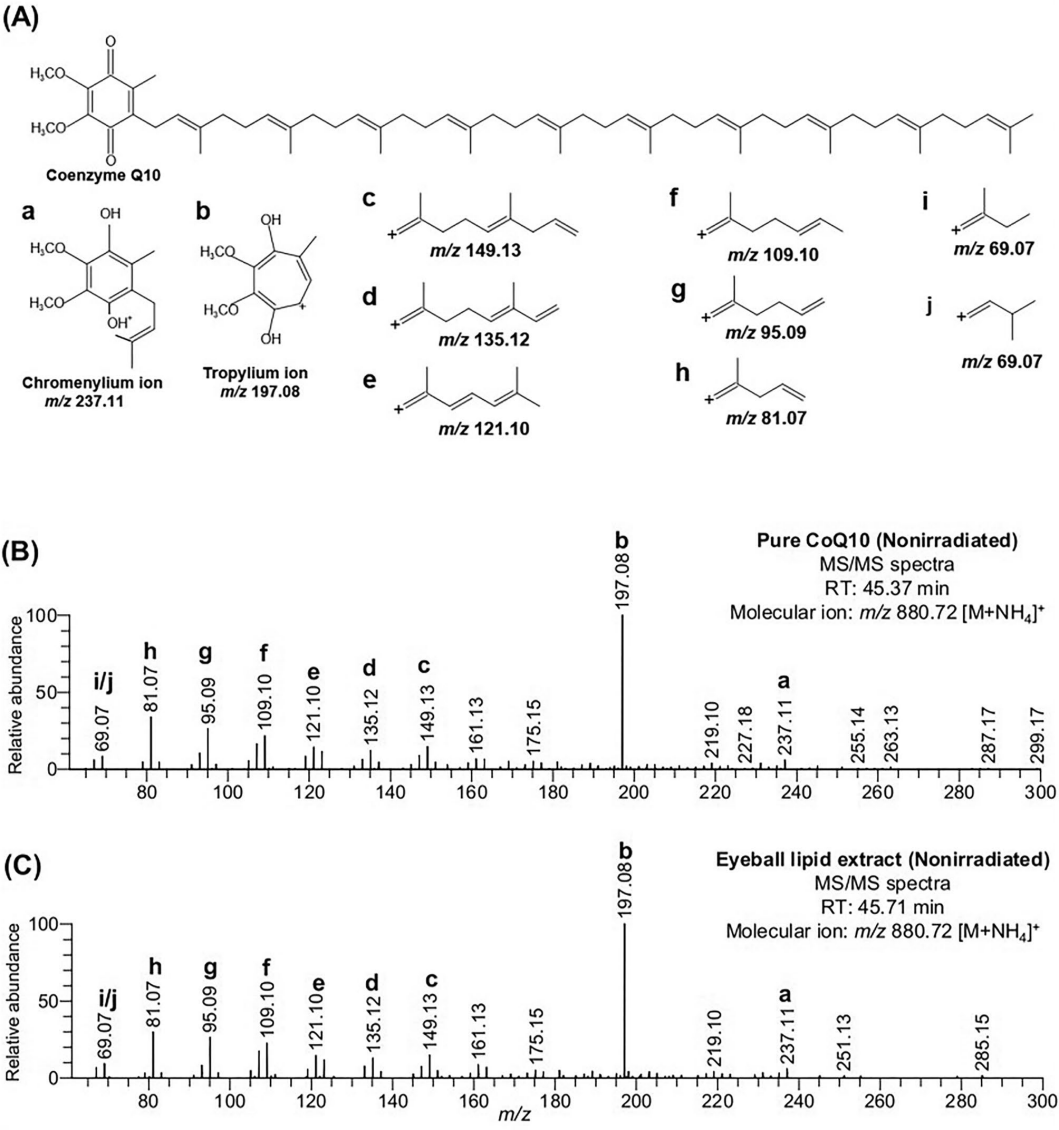
Structural analysis of ion at *m/z* 880.72. (**A**) The structure of natural CoQ10 (all-*trans* form) with known and possible fragment ions (a–j). (**B**) MS/MS spectra of nonirradiated pure CoQ10. (**C**) MS/MS spectra of the molecular ion at *m/z* 880.72 (tentatively assigned as [CoQ10 + NH_4_]^+^) were observed in nonirradiated eyeball lipid extract data. The fragments of CoQ10 shown in (**A**) were marked in the MS/MS spectra.

As already described, the LipidSearch™ assigned the *m/z* 863.69 and *m/z* 880.72 in the eyeball extract data as [M + H]^+^ and [M + NH_4_]^+^ adduct of CoQ10, respectively. To verify the assignment of CoQ10, we investigated the mass spectra and MS/MS spectra observed in eyeball extract and compared them with those of pure compounds. As is seen in Fig. S2, the ion at *m/z* 863.69 and *m/z* 880.72 were detected as the monoisotopic peak in both nonirradiated and irradiated eyeball extract. The nonirradiated pure CoQ10 (oxidized form and all-*trans*-isomer) was also detected at *m/z* 863.69 and *m/z* 880.72, which belong to [M + H]^+^ and [M + NH_4_]^+^ adducts, respectively (Fig. S2C). The isotopic distribution of these two ions in pure CoQ10 data was consistent with those of eyeball extract data (Fig. S2). Of the ions at *m/z* 863.69 and *m/z* 880.72, the latter showed a higher signal intensity in the mass spectra of both eyeball extract and pure CoQ10 (Fig. S2). In this study, we investigated the fragments of the molecular ion at *m/z* 880.72 to visualize the clearer MS/MS spectra. Interestingly, we observed similar fragmentation patterns between the spectra obtained from the eyeball extracts and pure CoQ10 (Fig. S3, Fig. 2). Upon tandem MS of CoQ (regardless of the length of isoprene tail), two fragment ions, namely tropylium ion (*m/z* 197.08) and chromenylium ion (*m/z* 237.11) are known to derive from the quinone head group, which is shared by all CoQ species^21–24^. Consistently, both of those fragments were seen in the MS/MS spectra of eyeball extract and pure CoQ10 (Fig. 2). The tropylium ion (*m/z* 197.08) was detected as the most abundant fragment in both MS/MS spectra (Fig. S3). In addition, a number of fragments (*m/z* 149.13, *m/z* 135.12, *m/z* 121.10, *m/z* 109.10, *m/z* 95.09, *m/z* 81.07, and *m/z* 69.07) that are associated with the isoprene chain were prominent in both MS/MS spectra (Fig. 2). These data made the assignment of CoQ10 unambiguous.

### CoQ10 in sunlight-irradiated eyeball extract was isomerized

**Figure 3:**
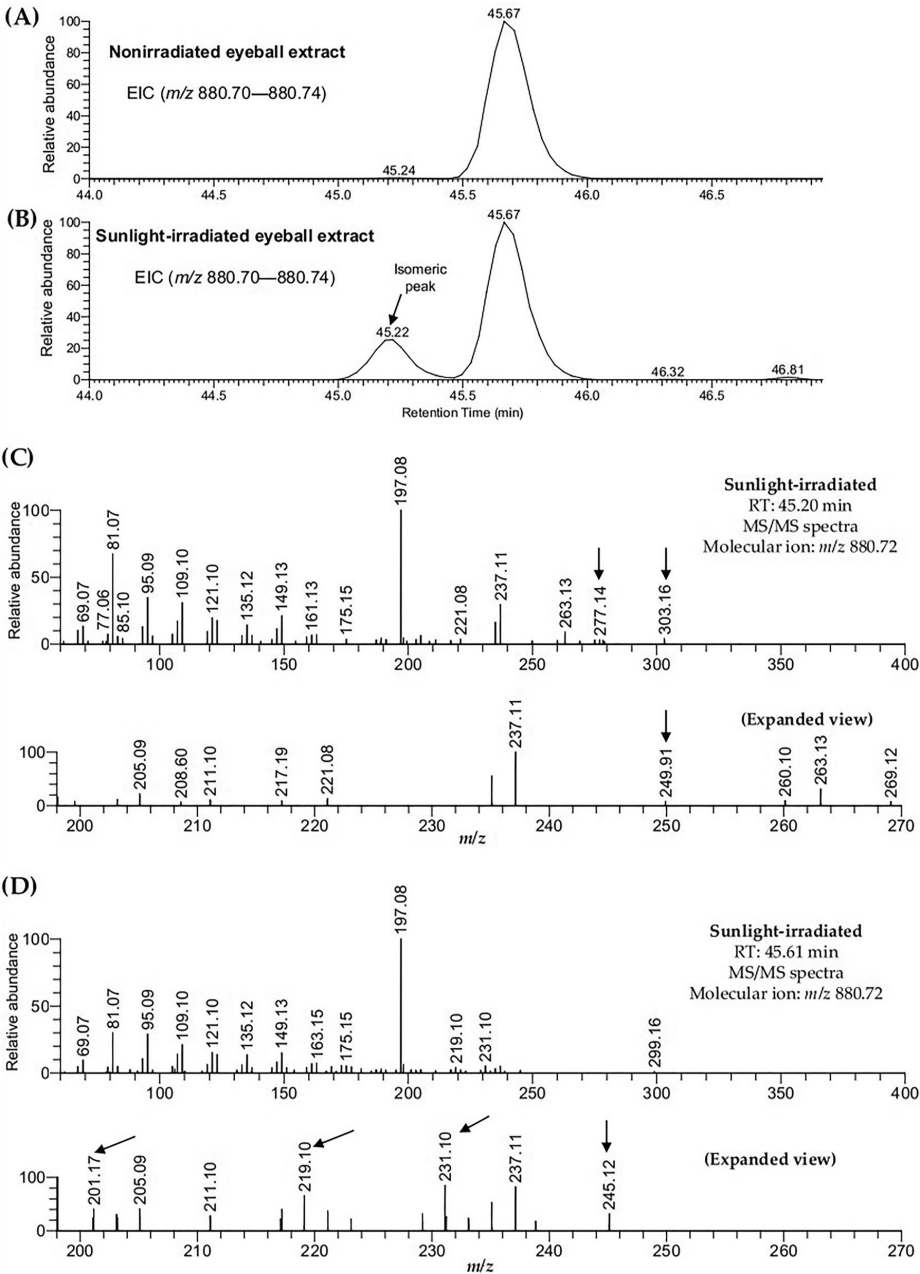
EIC and MS/MS spectra of coenzyme Q10 (CoQ10) in sunlight-irradiated eyeball extract. The EICs were observed in the (**A**) nonirradiated and (**B**) irradiated extract. MS/MS spectra of CoQ10 at (**C**) RT 45.20 min and (**D**) RT 45.61 min in the irradiated extract. Arrows indicate the fragmentations exclusively observed at RT 45.20 min or RT 45.61 min.

Next, we analyzed the extracted ion chromatogram (EIC) for the ion of *m/z* 880.72 of the eyeball extract data in order to investigate the isomeric CoQ10 molecules. Interestingly, we observed two distinct peaks at two different retention times (RT) in the irradiated sample (Fig. 3B), whereas a single peak was found in the nonirradiated one (Fig. 3A). For the EIC of CoQ10, the single peak in the nonirradiated sample and the right peak in the irradiated sample will be termed the “original peak” in this article. The MS/MS spectra (molecular ion at *m/z* 880.72) acquired at two RT (RT 45.20 min and RT 45.61 min) in the irradiated sample showed a similar pattern (Fig. 3) with some characteristic fragment ions (Fig. 3C,D). The fragment ions of *m/z* 249.91, *m/z* 277.14, and *m/z* 303.16 were exclusively observed at RT 45.20 (Fig. 3C). On the other hand, the characteristic fragment ions observed at RT 45.61 were *m/z* 201.17, *m/z* 219.10, *m/z* 231.10, and *m/z* 245.12 (Fig. 3D). These results indicated that CoQ10 in eyeball extract was isomerized by sunlight irradiation.

### Pure CoQ10 was isomerized along with altered physical properties by sunlight irradiation

**Figure 4:**
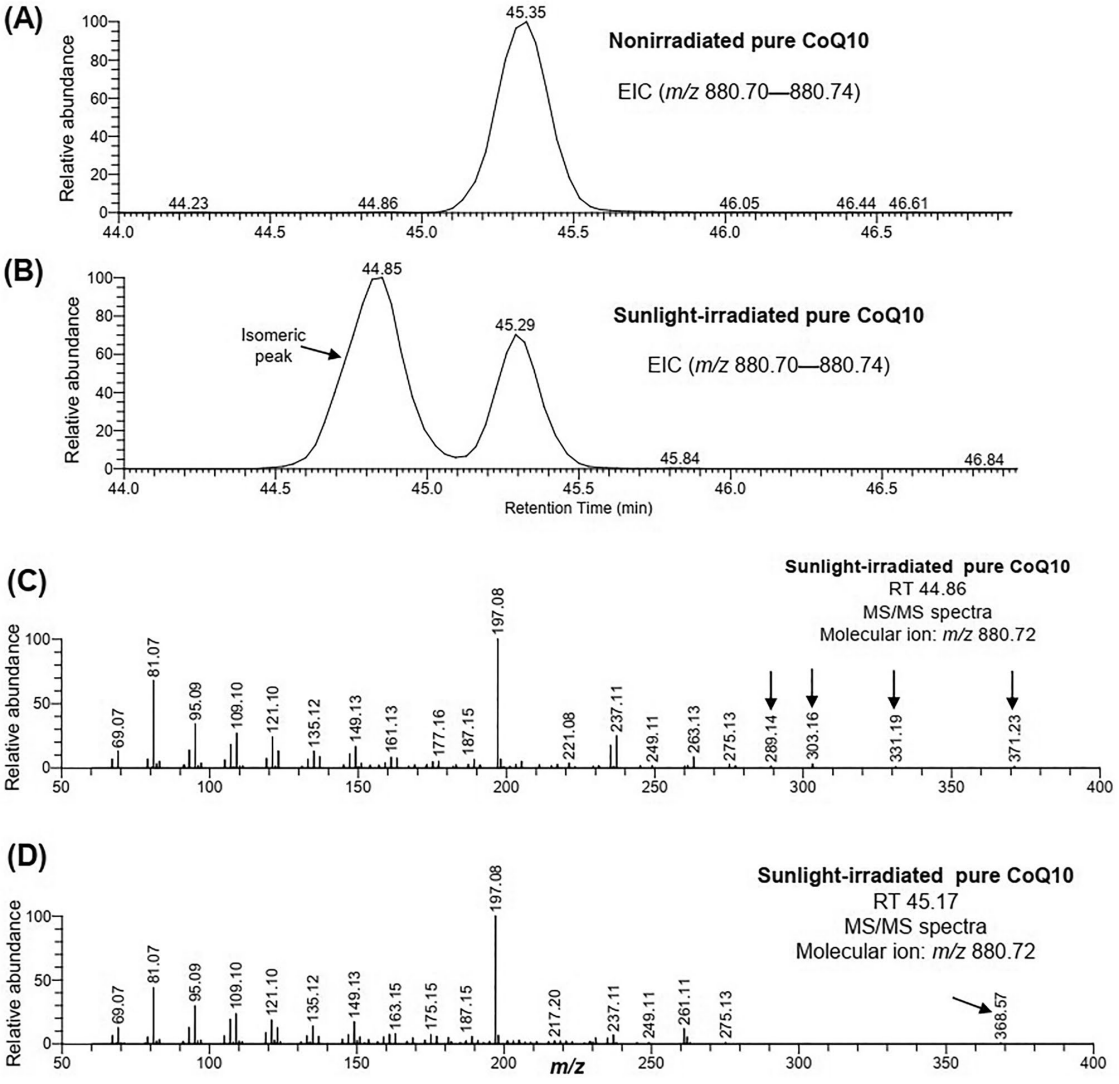
EIC and MS/MS spectra of pure coenzyme Q10 (CoQ10). EICs were observed in the (**A**) nonirradiated and (**B**) sunlight-irradiated samples. MS/MS spectra of CoQ10 at (**C**) RT 44.86 min and (**D**) RT 45.17 min in the sunlight-irradiated sample. Arrows indicate the fragmentations exclusively observed at RT 44.86 min or RT 45.17 min.

To confirm the photoisomerization of CoQ10 in the eyeball, we prepared the solution of the pure compound, irradiated it in the sunlight similarly, and then analyzed it by LC–MS/MS. As expected, a single peak at 45.3 min (excluding the peak which generally appears at the beginning due to the elution of highly hydrophilic compounds that do not bind to the column) was mainly observed in the TIC of the nonirradiated pure CoQ10 (Fig. S4A). On the other hand, in addition to the peak at 45.3 min, a number of peaks were observed in the TIC of sunlight-irradiated pure CoQ10 (Fig. S4B), indicating that the isomerization and/or any other phenomena (e.g., degradation) of some CoQ10 molecules has occurred. Similar to the results of eyeball extract, an additional peak (at RT 44.8 min) at the left of the original peak (RT 45.3 min) was observed in the EIC of CoQ10 in the irradiated pure sample (Fig. 4B). Interestingly, the left peak of CoQ10 showed a larger area than the right peak. It is also noteworthy that the isomeric peak (at RT 44.8 min) of CoQ10 was the most prominent among the additional peaks in the TIC of irradiated pure CoQ10 (Fig. S4, Fig. 4). The MS/MS spectra at two different RT showed a similar pattern with several characteristic fragment ions, which were exclusively observed at RT 44.86 min or 45.17 min (Fig. S5, Fig. 4C,D). These results indicated that pure CoQ10 was isomerized by sunlight irradiation. It is to be noted that CoQ10H2 was not detected in this data (Fig. S6), meaning that sunlight did not mediate the reduction of CoQ10 to CoQ10H2.

We also performed LC separations in conjunction with ion mobility spectrometry-mass spectrometry (LC-IMS-MS). Consistently, a difference in drift time of 0.05 ms was observed between the isomeric peaks of sunlight-irradiated pure CoQ10 (Fig. S7).

Next, we examined the physical properties of the nonirradiated and sunlight-irradiated CoQ10 through direct visualization and turbidity measurement. Before irradiation, the pure CoQ10 in Bligh and Dyer solvent produced a yellow-colored solution (Fig. S8A). Interestingly, the sunlight exposure turned the color of the solution from yellow to orange (Fig. S8B). The solutions were then dried and re-dissolved in 100% methanol, where we observed a cloudy and transparent solution of the nonirradiated and sunlight-irradiated CoQ10, respectively (Fig. S8C). Consistently, the turbidity of the nonirradiated solution in methanol was much higher than that of the irradiated solution (Table S2). These altered physical properties might be attributed to the isomeric CoQ10 produced upon irradiation.

### CoQ10 in sunlight-irradiated fresh eyeball was also isomerized

**Figure 5:**
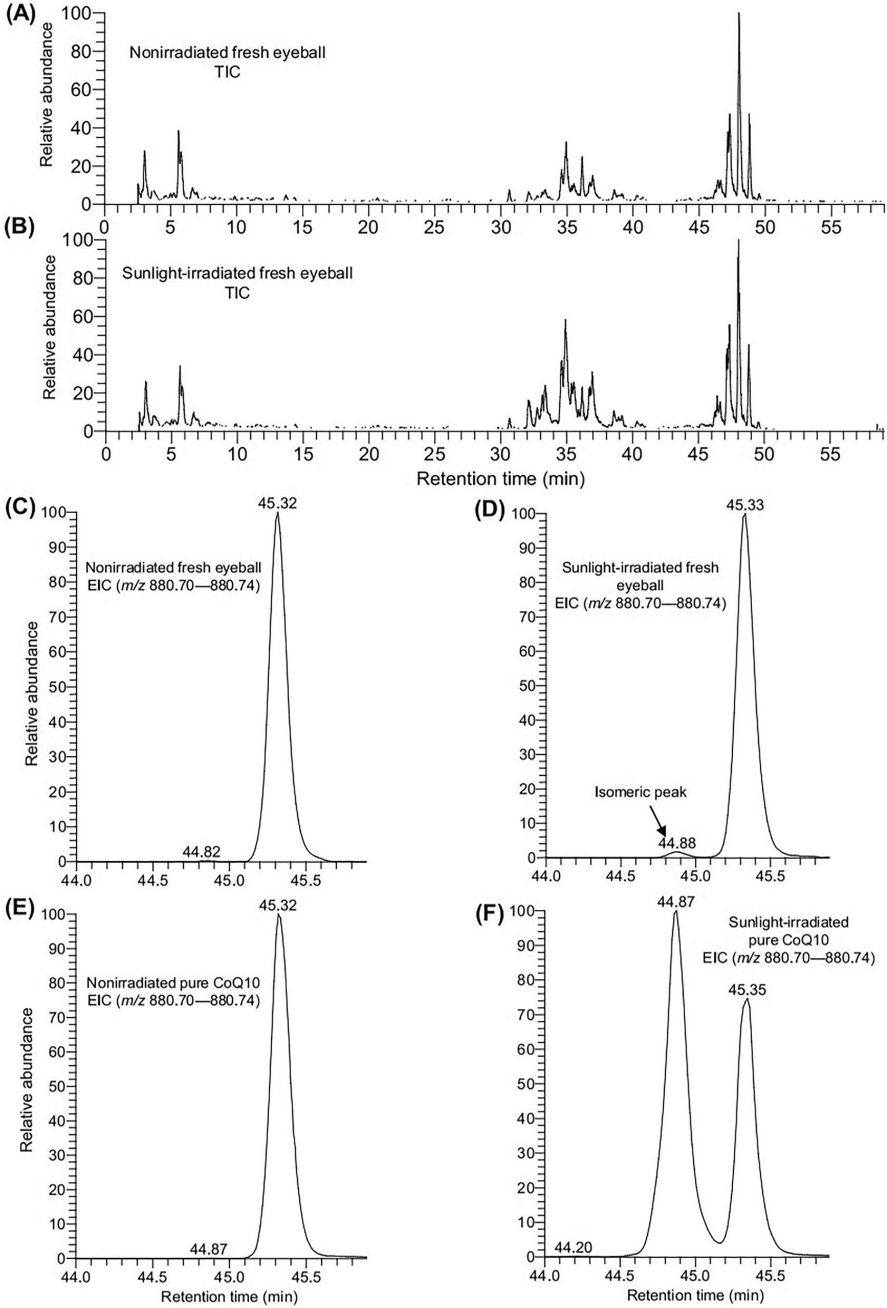
LC/MS data of fresh eyeball and pure CoQ10. TIC of (**A**) nonirradiated and (**B**) sunlight-irradiated fresh eyeball. The data shown here were obtained in positive ion mode. EIC of CoQ10 in (**C**) nonirradiated and (**D**) sunlight-irradiated fresh eyeball. EIC of CoQ10 in (**E**) nonirradiated and (**F**) sunlight-irradiated pure CoQ10.

Next, we investigated whether sunlight irradiation is capable of producing an isomer of CoQ10 in the freshly-prepared eyeball. For this purpose, the fresh eyeballs were irradiated in the sunlight, followed by lipid extraction and LC/MS analysis. Pure CoQ10 was also analyzed under the same conditions to confirm the consistency of RT. The TIC of nonirradiated and irradiated fresh eyeballs showed a similar pattern (Fig. 5A,B). Also, the EIC of CoQ10 in the nonirradiated fresh eyeball data showed a single peak (at RT 45.32), whereas the irradiated fresh eyeball data showed an additional peak (RT 44.88 min) at the left side of the original peak (Fig. 5C,D). The retention time was consistent with that of pure CoQ10 data (Fig. 5C–F). These results indicated that the CoQ10 in the fresh eyeball was also isomerized upon sunlight irradiation.

### Ultraviolet (UV) radiation mediated photoisomerization of CoQ10 rapidly

**Figure 6:**
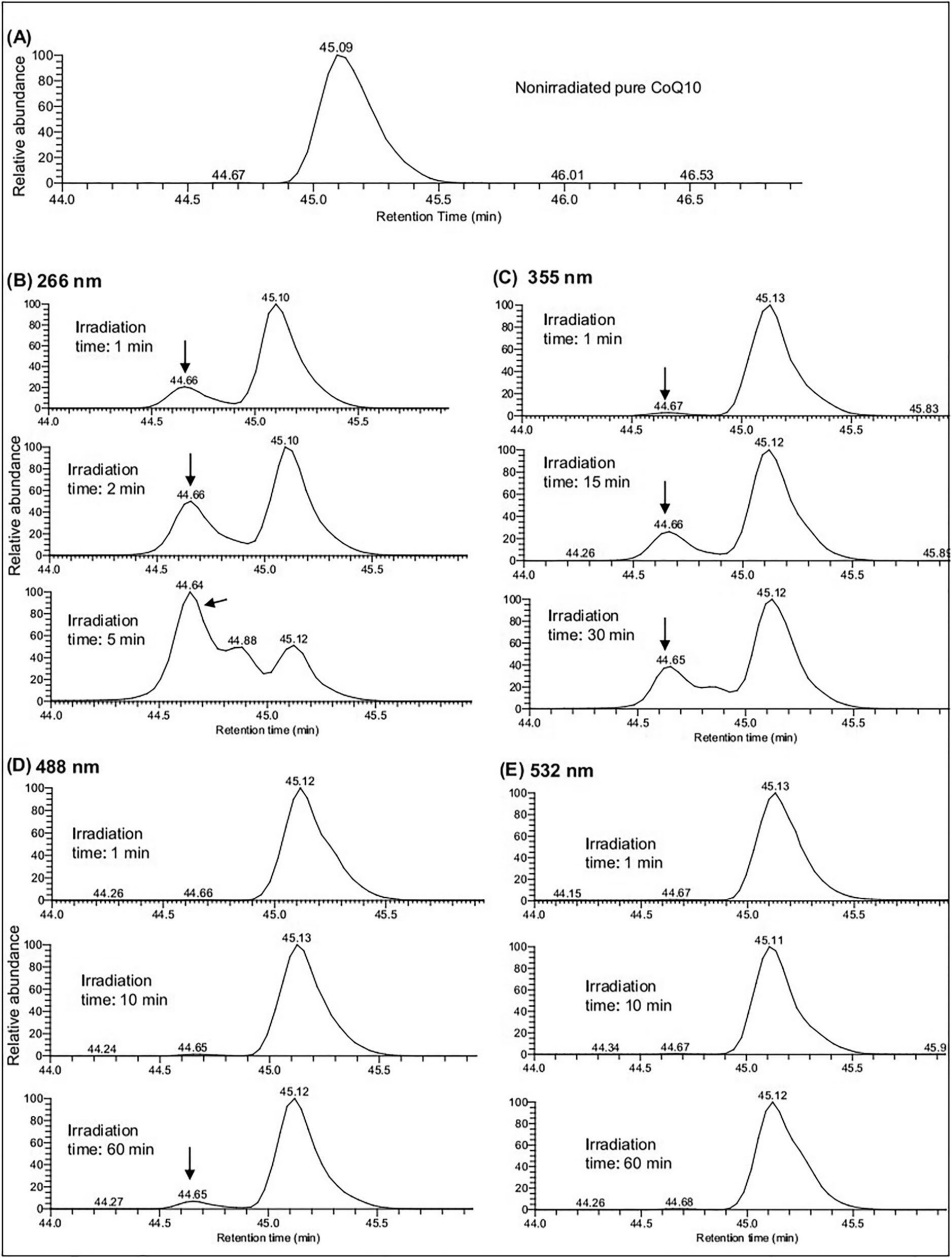
EIC (at *m/z* 880.70–880.74) of laser-irradiated pure coenzyme Q10. EICs were observed in (**A**) nonirradiated and (**B**–**E**) laser-irradiated pure CoQ10 at 266 nm, 355 nm, 488 nm, and 532 nm. Arrows indicate the isomeric peak (due to laser irradiation) of CoQ10.

Next, we irradiated pure CoQ10 solution with laser (UV and visible light) followed by LC/MS analysis in order to identify the range of the light spectrum that induces photoisomerization. Compared to the TIC of the nonirradiated pure CoQ10, the laser-irradiated pure CoQ10 showed a number of additional peaks (Fig. S9). Similar to the data in Figs. 3, 4, and 5, a single peak at RT 45.1 min was observed in the EIC of the nonirradiated sample (Fig. 6A). As expected, an additional peak at RT 44.6 min (left to the original peak at RT 45.1 min) appeared in the EIC of laser-irradiated pure CoQ10 solution (irradiated for 1 min, 2 min, and 5 min with 266 nm laser light) (Fig. 6B). A similar phenomenon was observed when the solution was treated for 15 min with a laser beam at 355 nm (Fig. 6C). The higher the duration of irradiation and the shorter the wavelength of UV light, the greater the isomeric peak area (at RT 44.6 min). On the other hand, 60 min of irradiation with visible light (488 nm but not 532 nm) was capable of producing an isomeric peak of CoQ10 (Fig. 6D). Consistently, we observed a decrease in the absorbance spectra of CoQ10 with the increase in irradiation time by UV light (Fig. S10). It is also to be noted that the isomeric peak was the most abundant among the additional peaks in the TIC, which was consistent with the data of sunlight-irradiated pure CoQ10. These results suggested that UV radiation was the main culprit that mediates the photoisomerization of CoQ10 rapidly (within minutes). The lower the wavelength, the higher the rate of isomerization (Fig. 6B–E).

## Discussion

In this study, we irradiated eyeball lipid extracts in the sunlight, followed by LC/MS and LC–MS/MS analyses in order to explore photoisomerizable lipid species. We identified the lipid species that were decreased in the irradiated eyeball extracts. Interestingly, CoQ10 showed the highest decrease (in terms of the fold change and *p*-value) among the surprising number of decreased lipid species (Fig. 1). Later, isomerization was found as the prominent cause underlying this decrease in CoQ10 level (Fig. 3). We also identified the UV radiation as the primary cause of this photoisomerization phenomenon (Fig. 6).

A variety of mechanisms protects against damage to the eye by light. For example, the cornea and the crystalline lens absorb almost all UV light while the visible light between 400 and 700 nm readily reaches the retina. In our study, although it required a much longer irradiation time by the visible light than UV radiation, the visible light was still capable of bringing about the isomerization of CoQ10 (Fig. 6). Consistently, CoQ10 in the sunlight-irradiated fresh eyeballs was also found to be isomerized (Fig. 5). Therefore, this study is believed to enhance our understanding of eye damage by sunlight exposure.

LC–MS is a powerful technique that allows the separation and subsequent detection and quantification of hundreds of analytes in a complex mixture of samples^25^. Besides, it has shown the capability of discerning isomeric compounds^26^, including lipid isomers. Previously, several researchers successfully resolved isomeric phospholipids, including those that have distinct fatty acyl positions (sn-1/sn-2)^27,28^ and *cis–trans* alkenes^29^. In this technique, a compound shows characteristic EIC (two or more peaks at different retention times belonging to the same species) for its isomers^8^. In our study, the EIC of CoQ10 in the irradiated samples showed two prominent peaks at two different RT that clearly indicated the isomerization.

Naturally, CoQ10 exists as an all-*trans* form, while chemical synthesis can produce a mixture of both *cis* and *trans* isomers^30,31^ (Fig. S12). In the current study, CoQ10 in the nonirradiated pure compound (all-*trans-*isomer) and the nonirradiated fresh eyeball were detected at the same RT (Fig. 5), indicating that CoQ10 in the eyeball naturally exists as an all-*trans*-isomer, hence, our data is in excellent agreement. It is to be noted that LC/MS analysis of frozen eyeball extracts and the pure CoQ10 were conducted on different days (i.e., the conditions were not identical), resulting in the inconsistency in the RT between Figs. 3 and 4.

The LC-IMS-MS is also a powerful method primarily used to separate and characterize isomers based on the drift time and the collision cross-section values. In our study, a small difference (0.05 ms) in the drift time was observed between the isomers. Advanced IMS techniques such as cyclic IMS would help to achieve better separation. Further analysis of irradiated eyeball extract along with the nonirradiated multiple isomers of CoQ10 (obtained by chemical synthesis or other means) by LC-IMS-MS is expected to help characterize the isomers in the eyeball. Nuclear magnetic resonance also can be an alternative to identify the unknown isomeric CoQ10.

CoQ10 exists as an oxidized or reduced form within the body. It is noteworthy that only the oxidized form was detected in both nonirradiated and irradiated eyeball samples in the current study. This might be due to the oxidation of CoQ10H2 during the lipid extraction and irradiation process since CoQ10H2 is known to be sensitive to oxidation^32^.

It is reported that CoQ10 and CoQ10H2 undergo degradation upon UV irradiation^33^. In addition to the isomeric peak, a number of additional peaks were observed in TIC and mass spectra of irradiated CoQ10 (Fig. S4, Fig. S6, Fig. S9), suggesting some other phenomena (that may include the degradation and dimerization of CoQ10) has also occurred upon irradiation. A further LC/MS study can be conducted to evaluate the degree of isomerization, breakdown or other phenomena of CoQ10 due to irradiation.

Light energy is known to mediate isomerization through several mechanisms, including thermal, photochemical, and mechanical effects^34,35^. Photoactive molecules are often isomerized by photochemical action as they can directly absorb light exciting the molecules to an upper electronic state. In photoisomerization events, the photon energy of the electromagnetic radiation often causes the rearrangement of *cis–trans* positions of organic compounds that contain a double bond in their structure. Typical examples of this phenomenon include the *cis–trans* interconversion of stilbene^36^ and azobenzene^37^. Besides, sunlight absorption by a solution results in the generation of free radicals^38^, which are known to mediate the geometrical isomerization of monounsaturated fatty acids^9^. CoQ10 has ten non-conjugated double bonds in its isoprene tail that can be a target of the free radicals. Eyes are known to generate free radicals upon light absorption^14^, indicating the possibility of free radical-mediated isomerization of CoQ10.

Isomerization often results in changes in the physical and chemical properties of isomers. Interestingly, CoQ10 was found to display altered physical properties, including color (yellow to orange) and solubility (increased solubility in methanol) upon sunlight irradiation (Fig. S8, Table S2). These changes might be attributed to the isomeric CoQ10.

Previously, Simeth et al. reported a photochromic derivative of CoQ where benzoquinone moiety was merged with two substituted thiophene moieties. The study demonstrated that light irradiation generates photoisomers of the photochromic derivative through an electrocyclic ring-closing reaction which is accompanied by a change in redox potentials of the benzoquinone moiety^39^. In contrast, we observed the photoisomerization of intact CoQ10 in eyeball extract, and the mechanism of this event is assumed to be different as the ring-closing reaction of the intact CoQ10 is not likely to occur. Hence, our findings are believed to contribute to the development of opto-lipidomics.

The CoQ10 plays an important role in the biological and medical fields. In human eyes, the consequences of CoQ10 deficiency are closely associated with aging-associated diseases, such as age-related macular degeneration and glaucoma^40^. Interestingly, topical application^41^ and dietary supplementation^42^ of CoQ10 were shown to have beneficial effects on patients with various ocular diseases. It has been reported that the rate of CoQ10 biosynthesis in the retina decreases with aging^43^, expecting the therapeutic role of CoQ10 in eye diseases.

Macular xanthophylls are well-known to prevent retinal photodamage by absorbing the blue light and removing the reactive oxygen species^44^. In addition, it has been shown that CoQ10 protects several parts of the eyes from light-induced damage. Instillation of CoQ10 as eye drops has been found to protect all retinal layers from apoptosis in mice^45^. Treatment with CoQ10 has been demonstrated to prevent the white light-induced apoptotic death of human lens epithelial cells in vitro by reducing oxidative stress and attenuating phototoxic effects on BAX and Bcl-2 expression^46^. It has also been demonstrated that CoQ10 reduces human corneal epithelial cell damage and vessel hyperemia induced by UVB irradiation^47^. Exogenous CoQ10 may play these protective roles through compensation for the loss of endogenous CoQ10_,_ as our current study revealed the light-induced photoisomerization that decreases the CoQ10 level. Hence, CoQ10 can have a potential application in ophthalmic surgeries. During these surgeries, the retina is exposed to a great deal of light stress. The administration of CoQ10 in the eye before such surgeries is expected to speed up the general healing process.

## Conclusion

The current study revealed the isomerization of CoQ10 in eyes due to sunlight irradiation. This study will enhance the understanding of the underlying mechanism of eye photodamage and potentially contribute to opto-lipidomics.

## Materials and methods

### Chemicals

Methanol (LC–MS quality), chloroform (HPLC quality), glacial acetate (HPLC quality), and water (Ultra-pure quality) were purchased from Wako Pure Chemical Industries (Osaka, Japan). A crystalline solid of pure CoQ10 (Chemical formula: C_59_H_90_O_4_, oxidized form, all*-trans*-isomer, purity: ≥ 98.0%, CAS No.: 303-98-0) was purchased from Cayman Chemical Company (Michigan, USA) and stored at − 20 °C until use.

### Samples

Pig (*Sus domesticus*) eyeball samples (n = 6) were obtained commercially (Funakoshi, Japan) and stored at − 80 °C until use. Another six pig eyeball (freshly prepared) were also obtained commercially (Tokyo-Shibaura-Zouki Co. Ltd., Japan) and stored at 4 °C for over a night.

### Preparation of eyeball extract and sunlight treatment

Approximately 10 g of each pig eyeballs (stored at − 80 °C) were homogenized with 40 mL of Milli-Q water by a blender, and the lipids were extracted by the modified Bligh and Dyer method^48^. Shortly, tissue lysate was mixed with 100 mL of methanol and 50 mL of chloroform and left for 10 min at room temperature. After that, an extra amount of chloroform (50 mL) and 0.28 M acetate (50 mL) was added and left for 30 min at room temperature. The mixture was then filtered using a glass filter funnel (filter pore size φ 100–120 μm). An equal volume of the lower layer of each eyeball extract was transferred into two clear glass bottles to obtain six paired samples, followed by the addition of 20 mL of 0.28 M acetate. One of the bottles from each paired samples was wrapped in aluminum foil, and then all of them were kept in the sunlight for over a day. After treatment, equal volume (8 mL) of the lower layer of each bottle was transferred into glass tubes, evaporated the solvents under vacuum conditions, and then stored at − 80 °C until further analysis.

### Analysis by LC/MS and LC–MS/MS

The stored lipids were re-dissolved in methanol, diluted further, and subjected to LC/MS analysis using Q Exactive™ Hybrid Quadrupole-Orbitrap™ Mass Spectrometer equipped with an electrospray ionization (ESI) source and attached to an Ultimate 3000 system (Thermo Scientific). Samples were injected at a volume of 10 μL followed by separation on Acclaim 120 C18 column (150 mm × 2.1 mm, 3 μm) (Thermo Scientific). Components of mobile phase A were prepared as follows: water–acetonitrile–methanol (2:1:1 v/v/v), 5 mM ammonium formate, and 0.1% formic acid. The composition of mobile phase B was as follows: acetonitrile–isopropanol (1:9 v/v), 5 mM ammonium formate, and 0.1% formic acid. For elution, the flow rate of the mobile phase was set at 300 μL/min. The total run time for the individual experiment was 70 min. A set of linear gradients starting at 20% solvent B was used and linearly increased to 100% B in 50 min, maintained at 100% B until 60 min, then decreased linearly to 20% B from 60 min to 60.1 min, and finished with 20% B for the last 10 min to equilibrate the column. Mass spectra were acquired from 2.5 to 60 min. Experimental conditions were optimized as follows: a capillary temperature of 250 °C; S-lens RF level of 50; and spray voltage of 3.5 kV in positive ion mode and 2.5 kV in negative ion mode. Mass spectra were acquired in a full-MS mode in the mass range of *m/z* 220–2000 with a mass resolving power of 70,000 (at *m/z* 200). For tandem mass spectrometry, a data-dependent MS/MS mode with a mass resolving power of 17,500 (at *m/z* 200) was used along with the LC/MS analysis. Xcalibur v3.0 Software (Thermo Scientific) was used for data acquisition.

### Analysis of sunlight-irradiated pure CoQ10 solution

A mixture of solvent (Water:0.28 M Acetic acid:Methanol:Chloroform = 4:5:10:10) was prepared to mimic the solvent ratio used for lipid extraction by Bligh and Dyer method. The lower layer of the mixture was used to prepare pure CoQ10 solution (1 mg/mL). An equal volume of the solution was transferred into six clear glass tubes to obtain three paired samples. One of the tubes from each pair were wrapped by aluminum foil followed by sunlight irradiation for over a day. After the treatment, the solvent of each tube was evaporated under vacuum conditions, re-dissolved in methanol and analyzed by LC/MS and LC–MS/MS using the same method described earlier.

The LC-IMS-MS analysis was also conducted using a hybrid quadrupole IMS orthogonal acceleration time-of-flight mass spectrometer (SYNAPT™ G2 High Definition Mass Spectrometry™ system, Waters, Milford, MA, USA) equipped with an ESI source and attached to an Acquity UPLC^®^ H Class PLUS (Waters, Milford, MA, USA). Data were acquired in positive ion mode in the mass range of *m/z* 100–1000 with a scan time and mass resolving power of 0.5 s and 20,000 (at *m/z* 200), respectively. Ionization conditions were optimized as follows: a capillary temperature of 150 °C, desolvation temperature of 450 °C, desolvation gas flow of 800 L/h, and a spray voltage of 4.0 kV. Helium was used as IMS gas. The TriWave parameters are given in Table S3. The other conditions that include LC column, column temperature, mobile phase, gradients, and flow rate were the same as that of LC/MS analysis.

### Examination of changes in physical properties

After sunlight treatment, we examined the color changes and transparency of the pure CoQ10 solution through direct visualization by the eye. The turbidity of the irradiated-pure CoQ10 solutions was measured by a transmitted/90-degree scattered light measuring system (TR-55 Turbidity Meter, KRK, Kasahara Chemical Instrument Corp., Saitama, Japan). Prior to the measurement, calibration was done using ultra-pure water and standard solutions (3.3 mg and 33 mg polystyrene particles per liter). 10 mL (60 µg/mL in methanol) of nonirradiated or irradiated pure CoQ10 solution was taken in a manufacturer-specified bottle and measured the turbidity as polystyrene turbidity (degree).

### Analysis of sunlight-irradiated fresh eyeballs

Three fresh eyeballs were wrapped in aluminum foil, and the other three were kept open, and then all of them were irradiated by sunlight for 8 h. Phosphate-buffered saline was used during the irradiation to prevent dryness. After irradiation, the muscles outside the eyeballs were trimmed off, followed by lipid extraction using the modified Bligh and Dyer method described earlier. The lower layer of the extract was collected, evaporated the solvents under vacuum conditions, and stored at − 80 °C until analysis. The samples were re-dissolved in methanol and subjected to LC/MS and LC–MS/MS analysis (the same method described earlier).

### Analysis of laser-irradiated pure CoQ10

In order to identify the light wavelength responsible for photoisomerization, we irradiated the standard CoQ10 (25 μg/mL) dissolved in ethanol by nanosecond pulsed laser (pulse width approximately 5 ns, 500 Hz repetition rate, average output 20 mW). The wavelength of the laser was 266 nm, 355 nm, 488 nm, and 532 nm, respectively. For 266 nm, we used the fourth harmonic of a nanosecond pulsed Nd:YAG laser (NL204, Ekspla, Lithuania). 355 nm was used for the third harmonic of a nanosecond pulsed Nd:YAG laser (NL204, Ekspla, Lithuania). 488 nm was used by pumping and oscillating a dye laser (dye: coumarin 500, CL-EGC, Ushio Optical Systems, Osaka, Japan) with the third harmonic of a nanosecond pulsed Nd:YAG laser (NL204, Ekspla, Lithuania). 532 nm was used for the fourth harmonic of a nanosecond pulsed Nd:YAG laser (NL204, Ekspla, Lithuania). The light exposure time was 1, 2, and 5 min for 266 nm, 1, 15, and 30 min for 355 nm, 1, 10, and 60 min for 488 nm, and 1, 10, and 60 min for 532 nm considering the absorbance of the sample at the irradiation wavelength (Table S4). After irradiation, the samples were diluted further and analyzed by LC/MS and LC–MS/MS (the same conditions described earlier). The nonirradiated sample was taken as a control.

The absorption of nonirradiated and laser-irradiated CoQ10 was also measured using an absorptiometer (UH5700, Hitachi High-Tech Corporation, Tokyo, Japan).

### Data analysis

LipidSearch™ software version 4.2.13 (Mitsui Knowledge Industry, Tokyo, Japan) was used for the tentative annotation and the relative quantification of lipid species in the six paired eyeball extracts. The LipidSearch™ parameter settings were as follows: database, HCD; search type, product_QEX; precursor tolerance, 5.0 ppm; product tolerance, 8.0 ppm; identification quality filters, A, B, and C. Quantification was done at *m/z* tolerance of ± 0.01 ppm with a retention time range from − 0.5 to 0.5 min. The identified lipid species among 12 samples were aligned with a retention time tolerance of 0.5.

For volcano plot preparation, the fold change of a lipid species was defined as an average area value of the irradiated samples divided by that of the nonirradiated samples. The horizontal axis and the vertical axis were expressed as log2(fold change) and − log10(*p*-value), respectively. Student’s *t* tests were conducted for statistical analysis and considered significant at *p* < 0.05 (with a fold change of ≥ 2.0 or ≤ 0.5).

For the dot plot, the fold change of an ion (associated with CoQ10) in a paired sample was calculated using the following formulae: area value observed in the irradiated sample/area value observed in the nonirradiated sample.

All of the spectra, TIC, and the EIC of LC/MS and LC–MS/MS data were analyzed by Xcalibur v3.0 Software (Thermo Scientific).

Assignment of CoQ10 was further confirmed by matching the mass spectra and MS/MS spectra with those of pure compounds and the previously published data^21–24^.

The data of LC-IMS-MS were analyzed by DriftScope™ Software, v. 2.1 (Waters, Milford, MA, USA).

## Supplementary Information

Supplementary Information 1.

Supplementary Information 2.

## Data Availability

The datasets generated during and/or analyzed during the current study are available from the corresponding author on reasonable request.
