# Supplementary material for: Coenzyme Q10 in the eye isomerizes by sunlight irradiation
Source: Sci Rep. 2022 Jul 15;12:12104. doi: 10.1038/s41598-022-16343-8 (PMC9287378; doi:10.1038/s41598-022-16343-8)
Supplement: Supplementary file 2 — Supplementary Information 2. [file 41598_2022_16343_MOESM2_ESM.pdf]

Supplementary file 2

Coenzyme Q10 in the eye isomerizes by sunlight irradiation

Md. Al Mamun[1], Md. Mahamodun Nabi[1], Tomohito Sato[1], Shuhei Aramaki[2], Yusuke Takanashi[1,3], Takumi Sakamoto[1], Kaito Hizume[1], Chikako Mori[1], Maiha Yasue[1], Masataka Ozaki[1], Ariful Islam[1], Tomoaki Kahyo[1], Makoto Horikawa[4], Yutaka Takahashi[1], Shigetoshi Okazaki[5], Kentaro Ohishi[6], Yu Nagashima[6], Keiji Seno[7], Yoshihiro Hotta[8], Mitsutoshi Setou[1,9,10,*]

[1]Department of Cellular & Molecular Anatomy, Hamamatsu University School of Medicine, 1-20-1 Handayama, Higashi-ku, Hamamatsu, Shizuoka, 431-3192, Japan.

[2]Department of Radiation Oncology, Hamamatsu University School of Medicine, 1-20-1 Handayama, Higashi-ku, Hamamatsu, Shizuoka, 431-3192, Japan.

[3]First Department of Surgery, Hamamatsu University School of Medicine, 1-20-1 Handayama, Higashi-ku, Hamamatsu, Shizuoka, 431-3192, Japan.

[4]Department of Molecular Biotechnology, Graduate School of Advanced Science of Matter, Hiroshima University, Higashi-Hiroshima, Hiroshima, 739-7763, Japan.

[5] HAMAMATSU BioPhotonics Innovation Chair, Institute for Medical Photonics Research, Preeminent Medical Photonics Education & Research Center, Hamamatsu University School of Medicine, 1-20-1 Handayama, Higashi-ku, Hamamatsu, Shizuoka, 431-3192, Japan.

[6]Institute for Medical Photonics Research, Preeminent Medical Photonics Education & Research Center, Hamamatsu University School of Medicine, 1-20-1 Handayama, Higashi-ku, Hamamatsu, Shizuoka, 431-3192, Japan.

[7]Department of Biology, Hamamatsu University School of Medicine, 1-20-1 Handayama, Higashi-ku, Hamamatsu, Shizuoka, 431-3192, Japan.

[8]Department of Ophthalmology, Hamamatsu University School of Medicine, 1-20-1 Handayama, Higashi-ku, Hamamatsu, Shizuoka, 431-3192, Japan.

[9]International Mass Imaging Center, Hamamatsu University School of Medicine, 1-20-1 Handayama, Higashi-ku, Hamamatsu, Shizuoka, 431-3192, Japan.

[10]Department of Systems Molecular Anatomy, Institute for Medical Photonics Research, Preeminent Medical Photonics Education & Research Center, 1-20-1 Handayama, Higashi-ku, Hamamatsu, Shizuoka, 431-3192, Japan.

***** Correspondence: setou@hama-med.ac.jp; Tel.: 053-435-2086; FAX: 053-435-2468

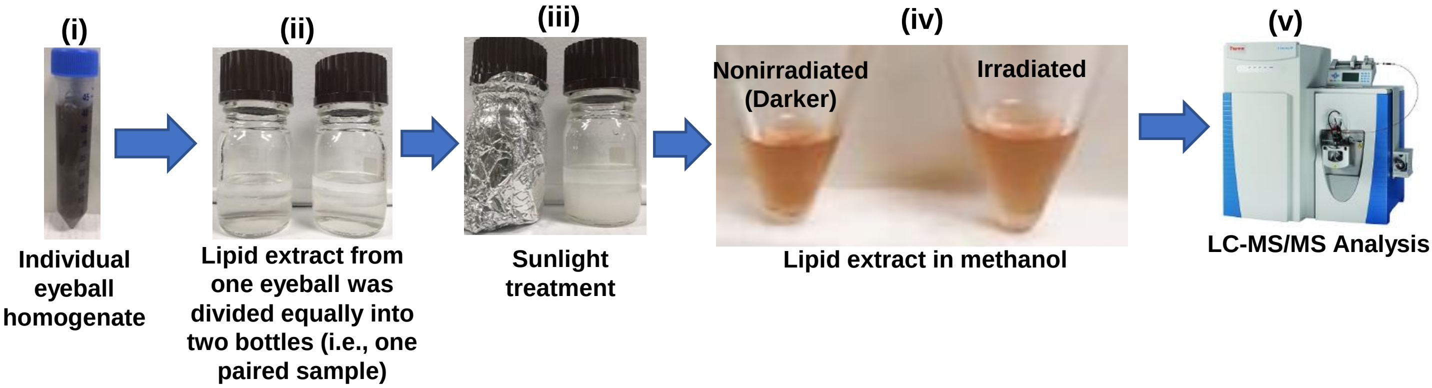

Figure S1: Experimental workflow for the exploration of photoisomeric lipids in pig eyeball. (i) The pig eyeballs were homogenized with Mili Q water by a blender. **(ii)** Lipids were extracted by the Bligh & Dyer method and equally aliquoted into glass bottles. **(iii)** Either was wrapped with aluminum foil (nonirradiated), and both bottles were illuminated using the sunlight for over a day. **(iv)** Extracts were transferred into glass tubes, evaporated under vacuum, and diluted with methanol. **(v)** Extracted lipids were subjected to LC/MS and LC-MS/MS analysis.

Table S1: Molecular formula, monoisotopic mass, and theoretical *m/z* values of coenzyme Q10 (CoQ10), coenzyme Q9 (CoQ9), and triglyceride(58:4).

Name of the molecules	Molecular formula	Monoisotopic mass	***m/z*** **(theoretical)**	
			**$[M+H]^+$**	**$[M+NH_4]^+$**
CoQ10 (oxidized), Ubiquinone-10 (CoQ10)	C59H90O4	862.68391	863.69174	880.71828
CoQ10 (reduced), Ubiquinol-10 (CoQ10H2)	C59H92O4	864.69956	865.70739	882.73393
CoQ9 (oxidized), Ubiquinone-9 (CoQ9)	C54H82O4	794.62131	795.62914	812.65568
CoQ9 (reduced), Ubiquinol-9 (CoQ9H2)	C54H84O4	796.63696	797.64479	814.67133
Triglyceride(58:4)	C61H110O6	938.83024	939.83807	956.86461

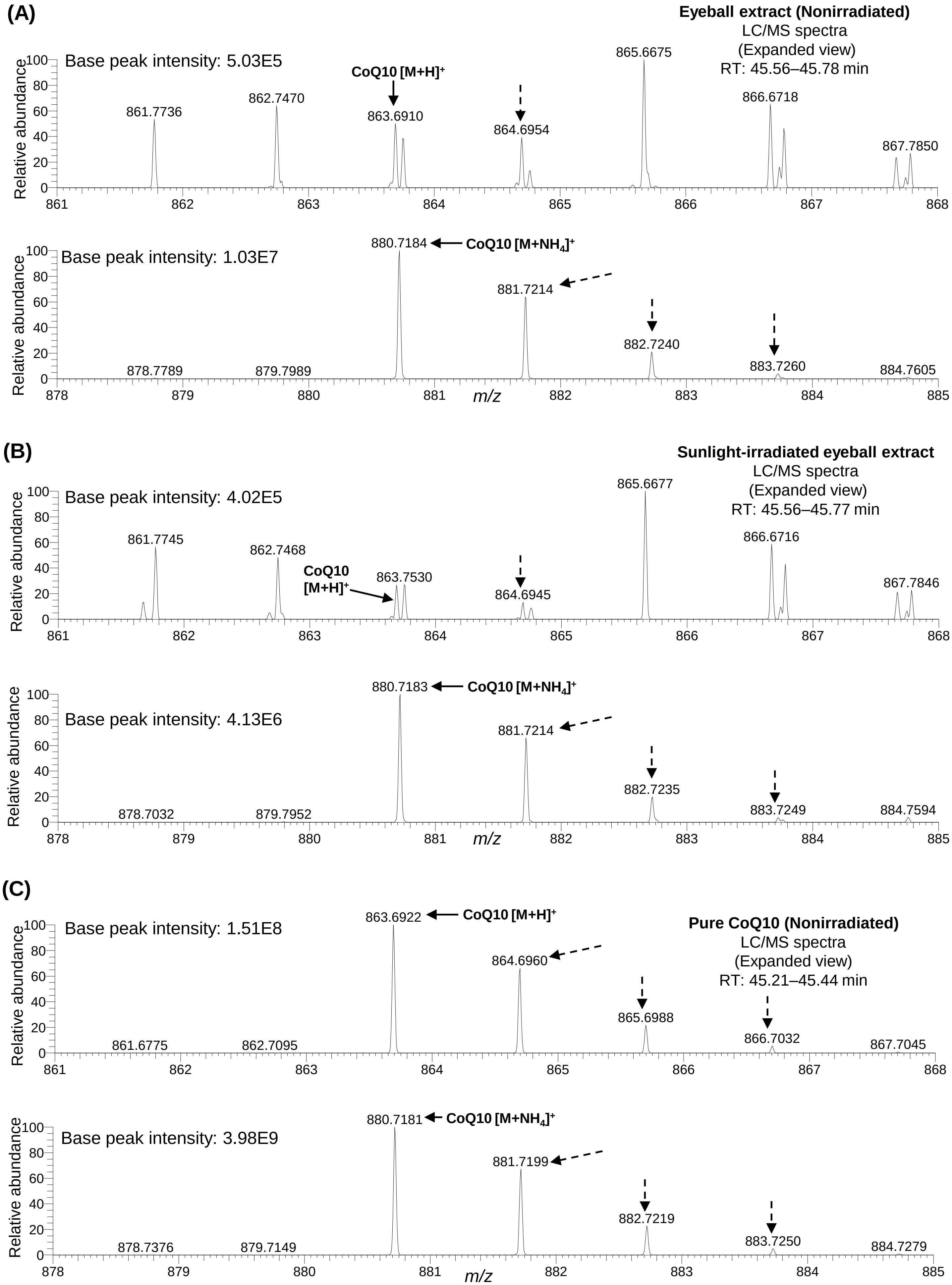

Figure S2: LC/MS spectra of CoQ10 (oxidized form) observed in (A) nonirradiated eyeball extract, (B) irradiated eyeball extract, and (C) nonirradiated pure CoQ10 (oxidized form) solution. The solid arrows and the dashed arrows indicate the monoisotopic peaks and other isotopic peaks of CoQ10, respectively.

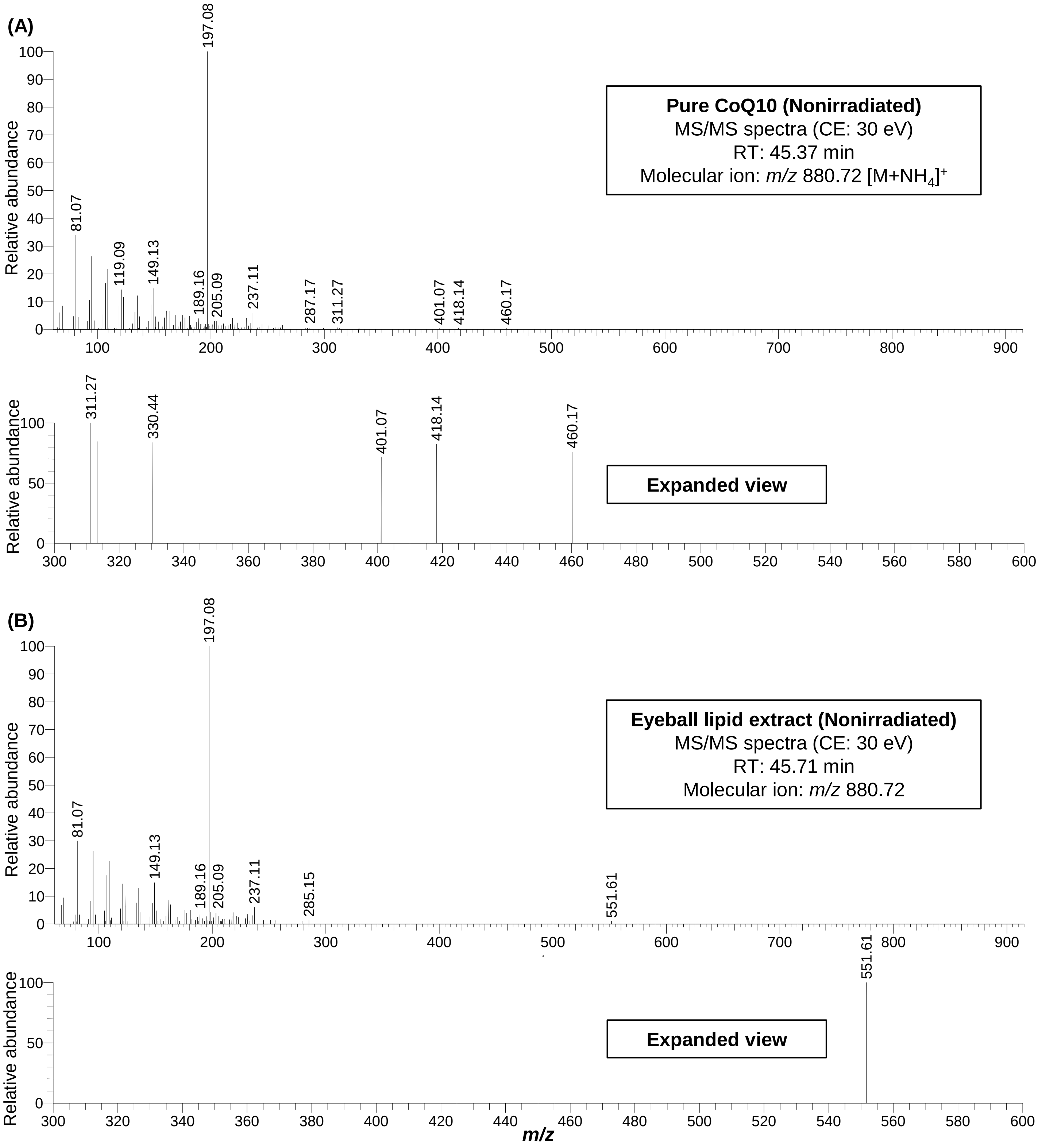

Figure S3: (A) Full MS/MS spectra of the molecular ion at *m/z* 880.72 observed in nonirradiated pure CoQ10. (B) Full MS/MS spectra observed in nonirradiated eyeball lipid extract. CE: Collision energy.

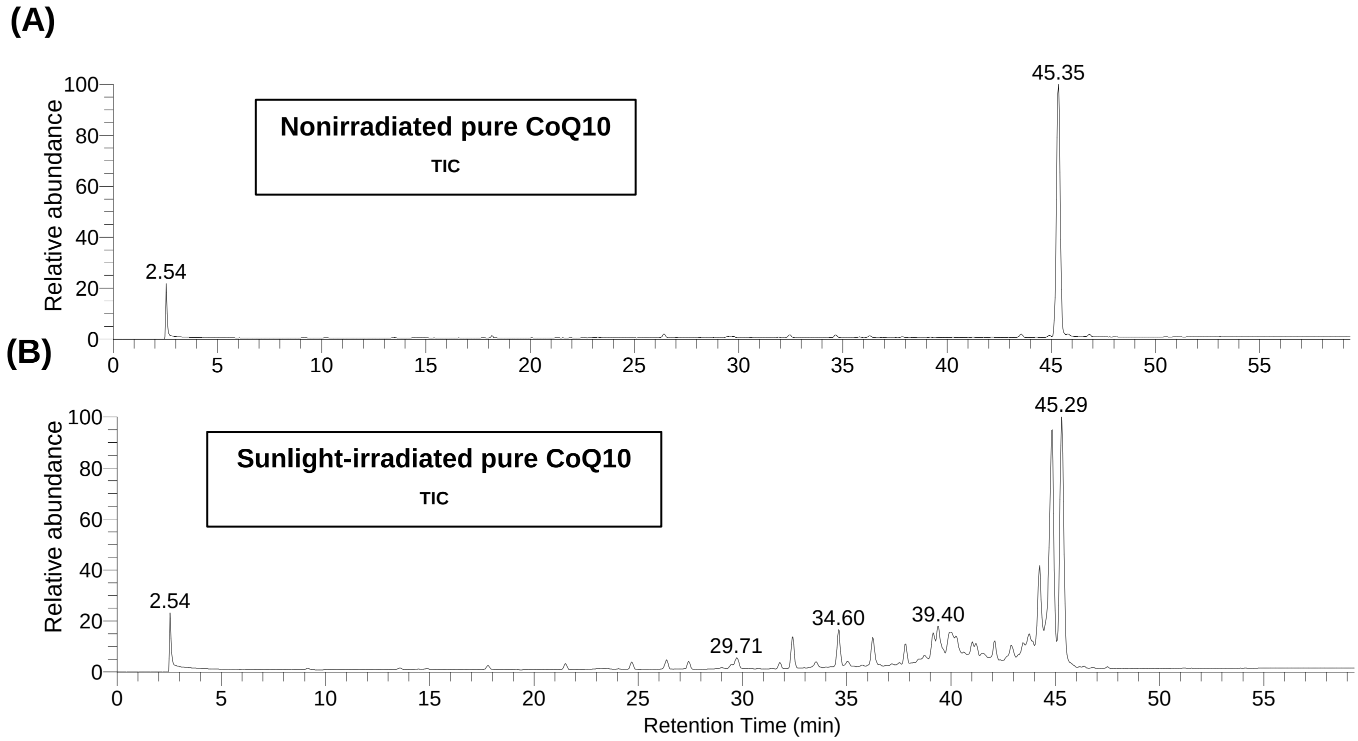

Figure S4: TIC of (A) nonirradiated and (B) sunlight-irradiated pure CoQ10. The data shown here were obtained in positive ion mode.

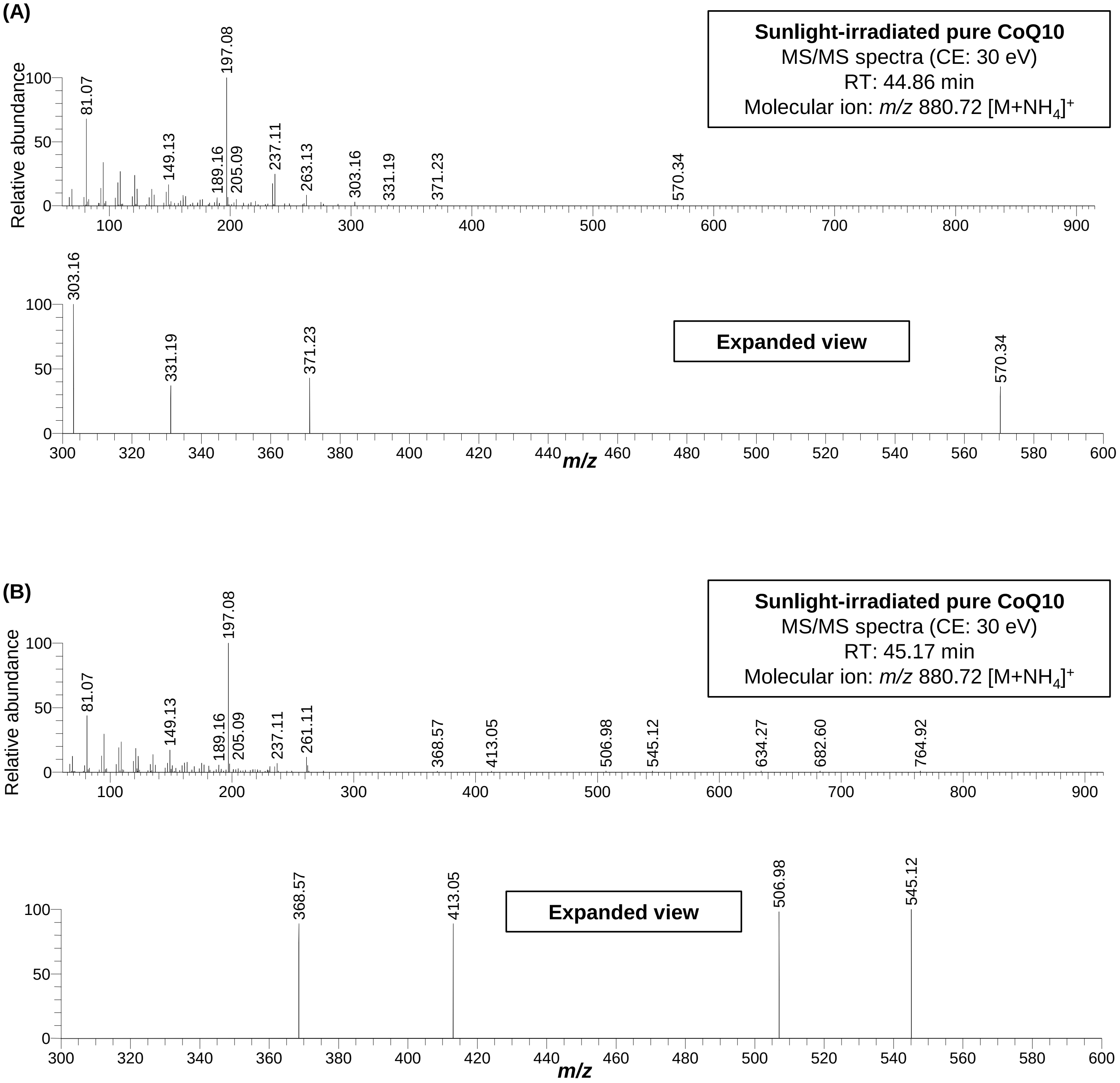

Figure S5: Full MS/MS spectra of the molecular ion at *m/z* 880.72 observed in sunlight-irradiated pure CoQ10. CE: Collision energy.

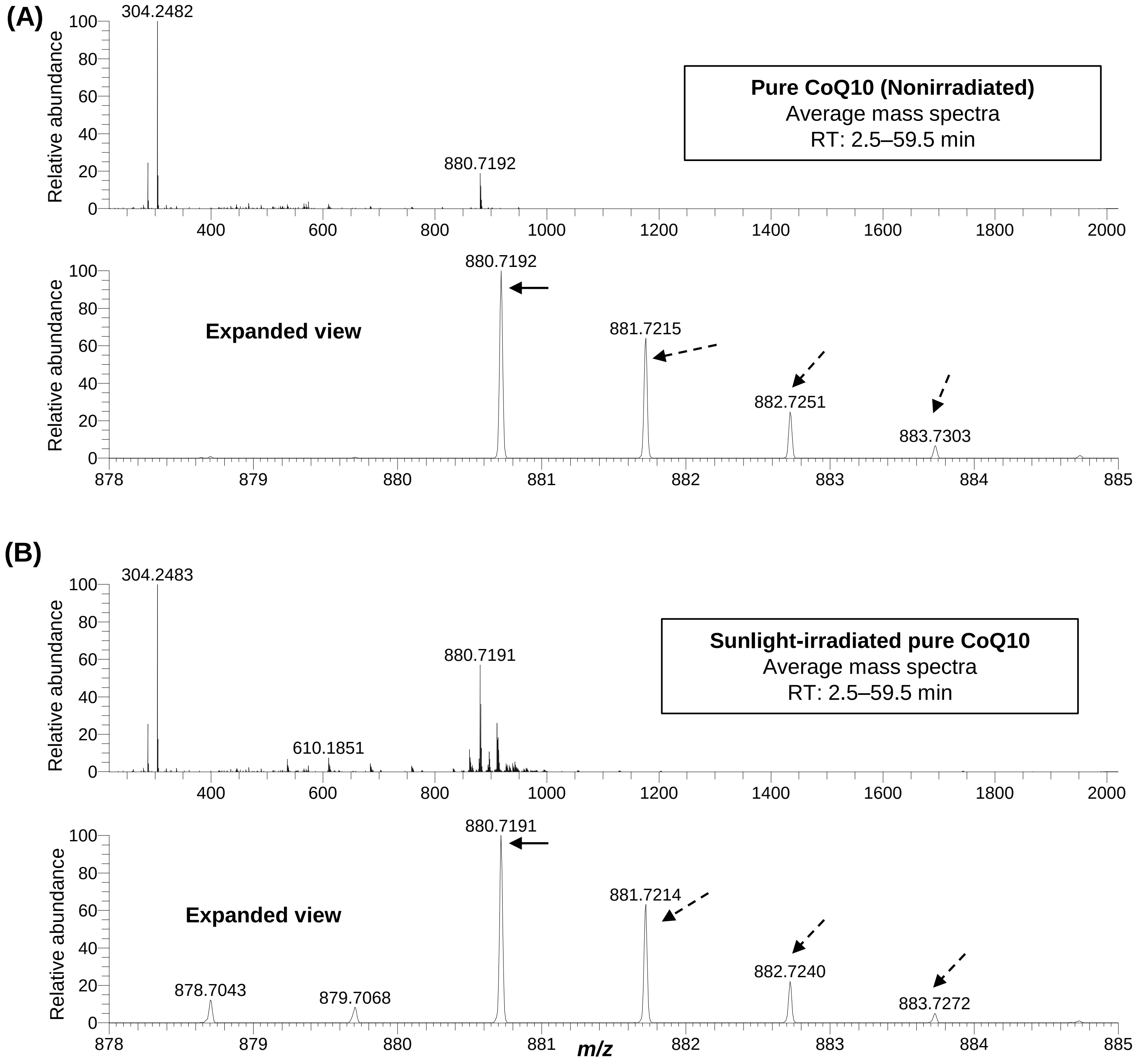

Figure S6: Average mass spectra (RT 2.5–59.5 min) of (A) nonirradiated and (B) sunlight-irradiated pure CoQ10. The solid arrows and the dashed arrows indicate the monoisotopic peaks and other isotopic peaks of CoQ10, respectively.

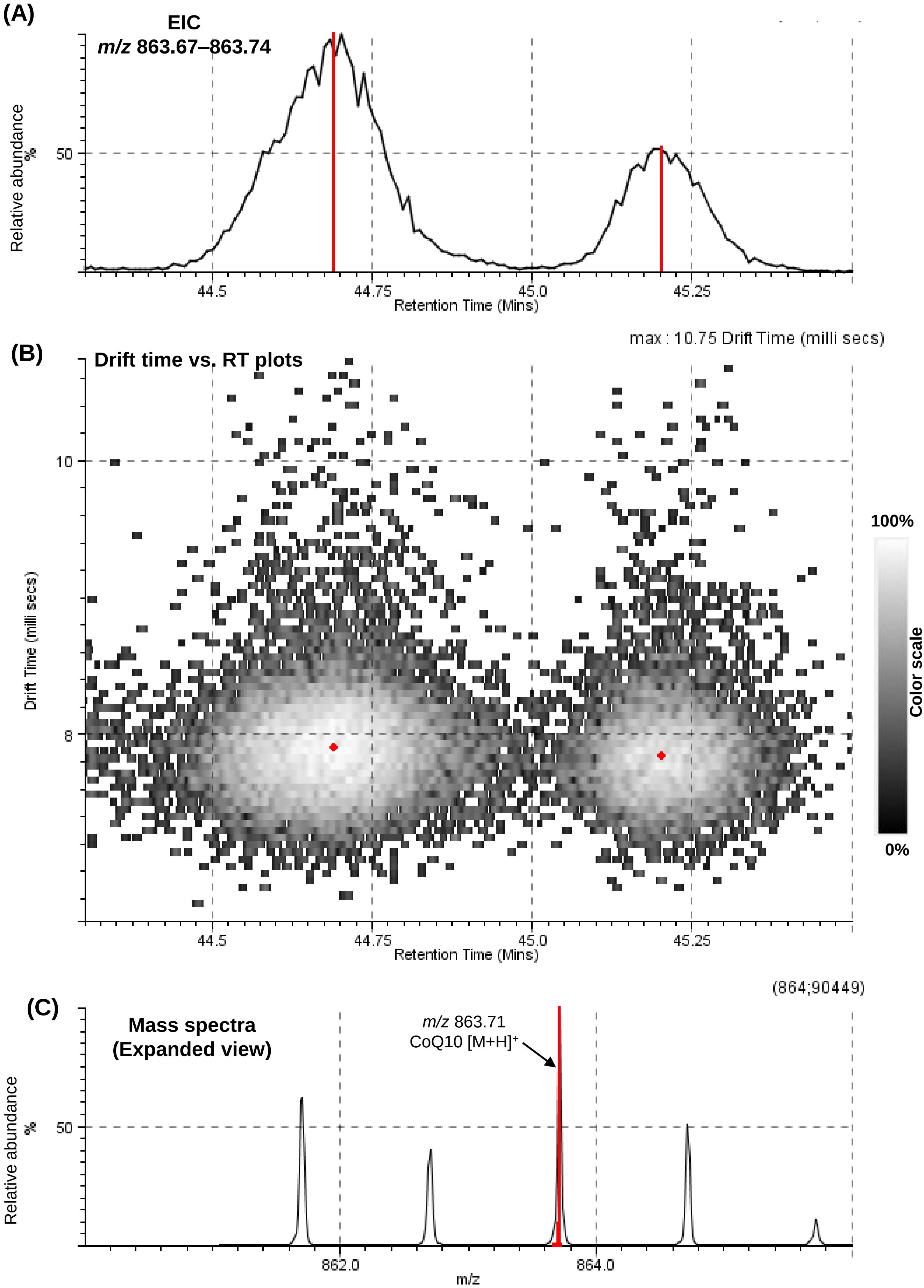

Figure S7: LC-IMS-MS data of sunlight-irradiated pure CoQ10. The red lines and the dots indicate the peak detection at the intensity threshold of 1,000.

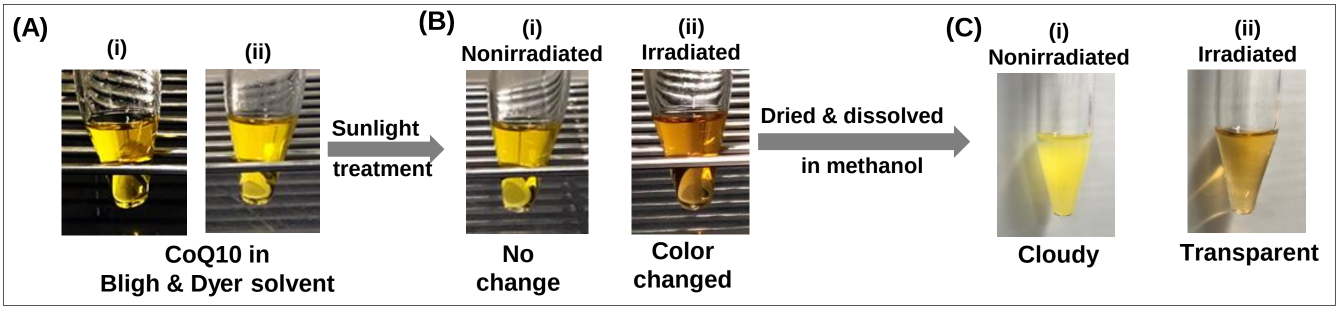

Figure S8: Effects of sunlight treatment on pure CoQ10 solution. Sunlight exposure changed the color of the pure CoQ10 solution. Solubility in methanol was increased after the irradiation.

Table S2: The turbidity of pure CoQ10 solution in methanol

Sample information	Turbidity (degree)			
	Pair 1	Pair 2	Pair 3	Mean
Nonirradiated pure CoQ10 in methanol	22.3	22	22.8	22.4
Sunlight-irradiated pure CoQ10 in methanol	0.0	0.0	0.0	0.0

Table S3: TriWave parameters

**TriWave Parameters**	**Trap**	**IMS**	**Transfer**
Wave velocity (m/s)	700.0	400.0	130.0
Wave Height (V)	5.0	37.0	10.0

Table S4: Light absorption rate of pure coenzyme Q10 solution in ethanol.

Wavelength of laser radiation (nm)	Duration (min)	Absorption rate (%)
256	1	54
355	15	4
488	60	1
532	180	0.3

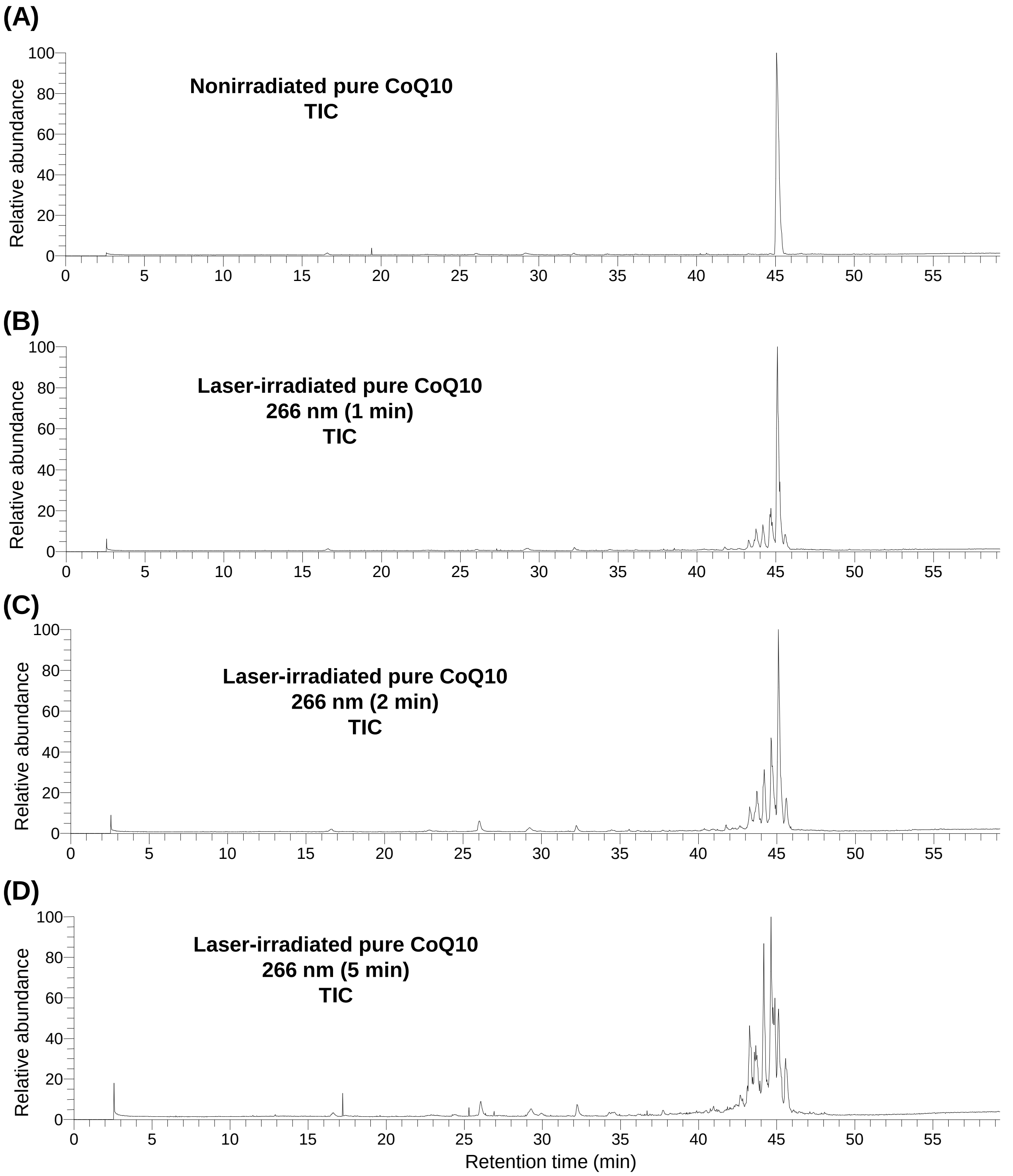

Figure S9: TIC of (A) nonirradiated and (B–D) laser-irradiated pure coenzyme Q10 solution at 266 nm. The data shown here were obtained in positive ion mode.

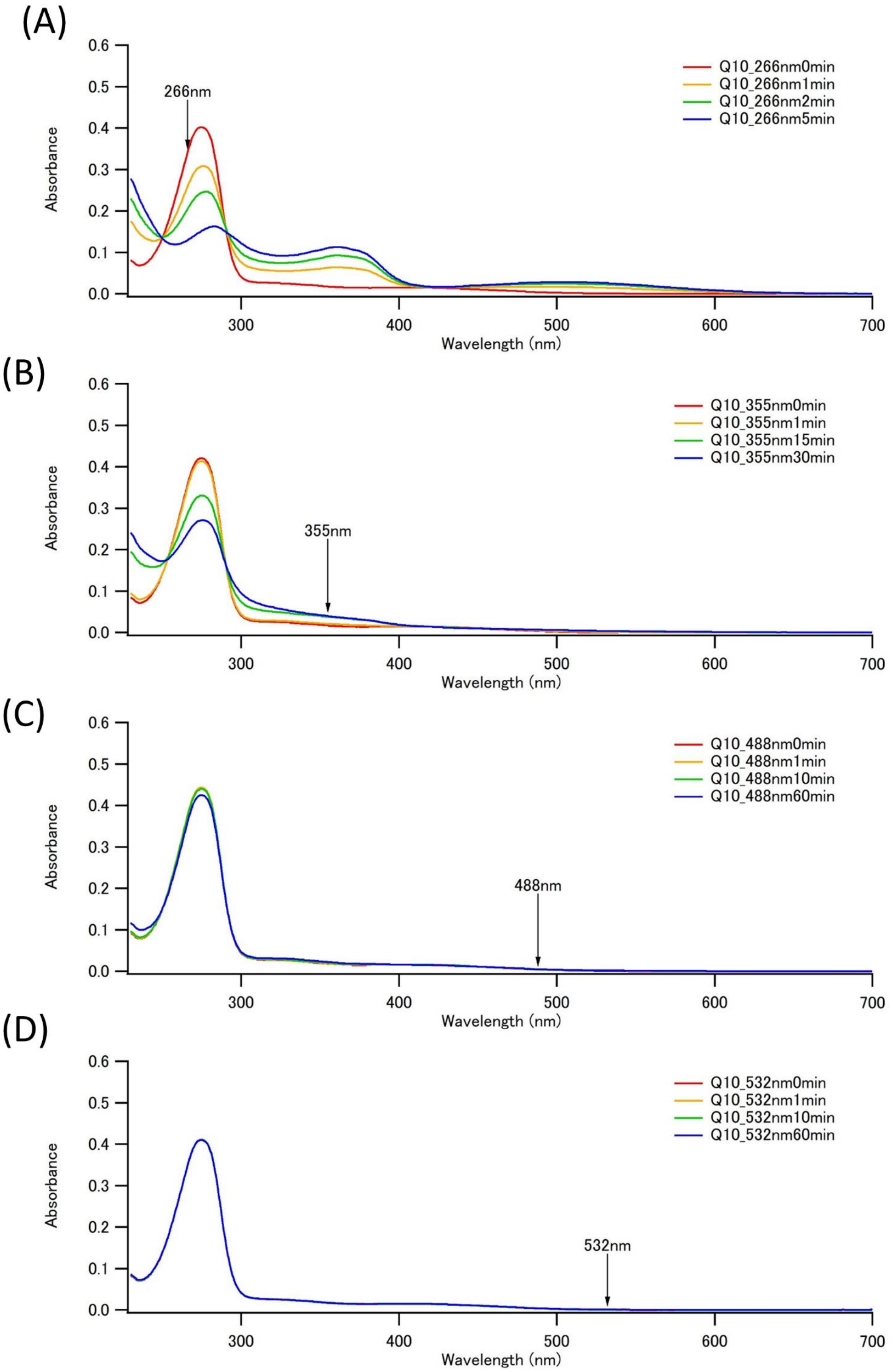

Figure S10: Absorbance spectra of the laser-irradiated pure coenzyme Q10 solution. The individual solution was irradiated with laser light at (A) 266 nm, (B) 355 nm, (C) 488 nm, and (D) 532 nm for several time periods and then subjected to absorbance measurements. The arrows indicate the wavelength used for the absorbance measurements.

Fresh eyeballs wrapped in aluminum foil | Fresh eyeballs kept open

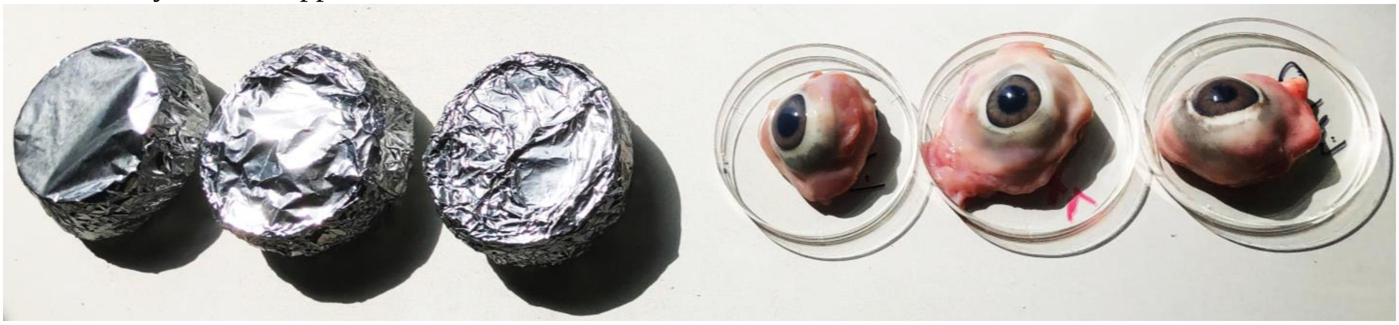

Figure S11: Optical images of fresh eyeballs kept in the sunlight

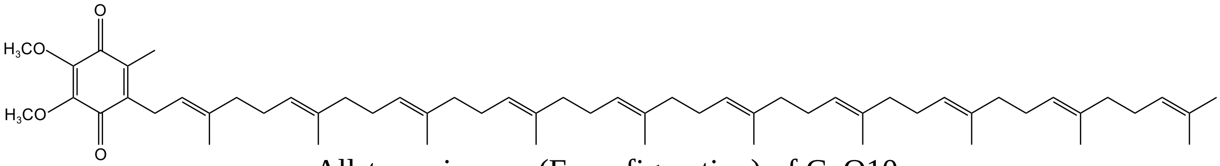

All-*trans*-isomer (E-configuration) of CoQ10
(natural CoQ10)

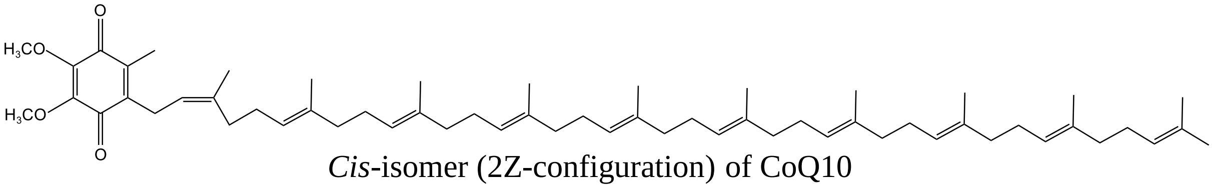

Cis-isomer (2Z-configuration) of CoQ10

Figure S12: Structure of natural CoQ10 and its potential isomeric form.
